# Cost-benefit ratio of anthelmintic treatment and its comparative efficacy in commercial dairy farms

**DOI:** 10.3389/fvets.2022.1047497

**Published:** 2022-11-16

**Authors:** Muhammad Rashid, Naveed Zahra, Amna Chudhary, Tauseef Ur Rehman, Muhammad Tahir Aleem, Abdulaziz Alouffi, Aymen Mohammed, Muhammad Imran Rashid, Muhammad Ehsan, Muhammad Irfan Malik, Ghulam Hussain Dilber, Amir Bakhsh, Mashal M. Almutairi

**Affiliations:** ^1^Department of Parasitology, Faculty of Veterinary and Animal Sciences, The Islamia University of Bahawalpur, Bahawalpur, Pakistan; ^2^Department of Livestock Management, Faculty of Veterinary and Animal Sciences, The Islamia University of Bahawalpur, Bahawalpur, Pakistan; ^3^College of Life Sciences, Shaanxi Normal University, Xi'an, China; ^4^MOE Joint International Research Laboratory of Animal Health and Food Safety, College of Veterinary Medicine, Nanjing Agricultural University, Nanjing, China; ^5^King Abdulaziz City for Science and Technology, Riyadh, Saudi Arabia; ^6^Division of Molecular Therapeutics and Formulation, School of Pharmacy, University of Nottingham, Nottingham, United Kingdom; ^7^Department of Parasitology, University of Veterinary and Animal Sciences, Lahore, Pakistan; ^8^Department of Theriogenology, Faculty of Veterinary and Animal Sciences, The Islamia University of Bahawalpur, Bahawalpur, Pakistan; ^9^Livestock and Dairy Development Department, Government of Punjab, Punjab, Pakistan; ^10^Department of Pharmacology and Toxicology, College of Pharmacy, King Saud University, Riyadh, Saudi Arabia

**Keywords:** anthelmintic, efficacy, cattle, buffaloes, heifers, cost-benefit ratio

## Abstract

Intestinal parasitic infection is one of the major challenges in obtaining optimal production and maintaining the health and welfare of all animals including cattle and buffaloes. Anti-parasitic treatments appear to be a reliable countermeasure. However, the effectiveness and selection of suitable anthelmintics require situational assessments in a given locality. In the current study, the efficacy and impact of benzimidazole (albendazole) were assessed in a total of 400 (100 each) on the performance of buffaloes, buffalo-heifer, cattle, and cattle-heifers at two commercial dairy farms in the Province of Punjab, Pakistan. Additionally, the cost-benefit ratio was calculated by assessing the inputs (medication, feed, and labor cost) and outputs (milk and weight gain). The qualitative and quantitative examination of helminth eggs in each type of animal indicated a prevalence of 73.3, 78.3, 76.6, and 85.0% in cattle, cattle-heifers, buffaloes, and buffaloes-heifers, respectively. Specifically, a highest rate (10.0–13.3%) of *Haemonchus* sp. infection was only observed in cattle and heifers, while *Fasciola* sp. infections (10.0–11.6%) were the most often found species in buffaloes and heifers. The highest anthelmintic impacts (egg per gram of feces, *p* < 0.001) were observed on day 14 post-medication. Until 60 days of post-anthelmintic treatment, an average increase of 0.8 and 0.7 L in milk production per day in cattle and buffaloes, respectively while a total of 11.45 and 9.45 kg body weight were noticed in cattle-heifer and buffaloes-heifer, respectively. Cumulative cost-benefit analysis indicated a positive correlation between treated and non-treated animals. These findings reiterate the importance of anthelmintic drugs in reducing the impacts of parasites on the productivity, health, and well-being of an animal under high infection challenges.

## Introduction

Livestock plays a major role in the economy of Pakistan; it contributes to 61.9% of the total agriculture revenue and shares 14.0% of Pakistan's gross domestic product (1). To supply cheap, high-quality and surplus milk, meat, and meat products for human consumption, farmers are favoring exotic highly productive breeds over local animal breeds (2). As low as 30–35 million people are engaged in the livestock sector in Pakistan, highlighting the potential and dependency of the economy on livestock (3). The major constraint to sustainable animal production comes from several diseases (4–13). Helminth infections not only reduce productivity but also compromise the quality of food. Parasitized animals reduce live weight gain, increased age of puberty, low productivity, and higher susceptibility to other pathogens; collectively contributing to severe economic losses to stakeholders (14, 15). Infection with various species of parasites in cattle and buffaloes is a growing global problem (16–29). The most prevalent cattle helminths include *Strongylus* sp., *Paramphistome* sp., *Strongyloides* sp., *Moniezia* sp., *Toxocara vitulorum, Trichuris* sp., *Fasciola* sp., and *Bunostomum* sp. (30). Depending upon animal rearing standards, resistance against parasites, and biosecurity measures, the prevalence of helminth infections is variable and ranges in the world from 0.78 to 84.1% (31). This prevalence ranges in Pakistan from 33.68 to 51.0% (32). As a countermeasure, broad-spectrum anthelmintic drugs such as albendazole and Ivermectin were used to significantly improve the health and well-being of animals which then favorably impact the productivity and standards of animal-origin food and food products (33). The recorded increment of milk production upon post-anthelmintic medication was estimated to be 0.42 L per day (34). The efficacy of albendazole against *Ostertagia ostertagi* ranges from 84.9 to 99% depending upon the developmental life stages of the parasite (35). Basically, anthelmintic drugs are effective in controlling parasitic infections, but excessive use of anthelmintics (under doses and narrow spectrum drugs) induced an increase in the resistant parasite population (36).

The present study was conducted to evaluate the effect of anthelmintic treatment on milk production and live weight gain in cattle, buffaloes, and their heifers. Moreover, the efficacy of the drug was monitored to ascertain existing anthelmintic resistance and a cost-benefit ratio was performed.

## Materials and methods

### Study area and sampling

The study was carried out during the spring season on two private dairy farms, consisting of buffaloes from the Sheikhupura district and cattle from the Rajanpur district of Punjab Province, Pakistan. A total of 400 samples (100 each) from cattle, cattle-heifer, buffaloes, and buffalo-heifer were collected for this study.

### Classification of animals

Animals were selected based on the following criteria: (i) Adult cattle and buffaloes were in the first trimester of lactation. (ii) Age of cattle-heifers and buffalo-heifers ranged from 5 to 8 months. (iii) Animals were not dewormed during the last 3 months prior to the study. (iv) Animals' eggs per gram (EPG) ≥ 200 in feces were included in the treated and control group (37). A total of 60 animals for each group of cattle and cattle-heifer (Holstein Friesian); and buffaloes and buffalo-heifers (Nili Ravi breed) were included for anthelmintic treatment to proceed with the present study. Live body weight was estimated by taking the girth circumference in centimeters (cm) with measuring tape and compared it with tables (http://bairnsley.com/Weight%20by%20Girth.htm) (37). The average adult buffalo and cattle weights were 500 and 550 kg, and buffalo-heifer and cattle-heifers were 90 and 150 kg, respectively. All animals at each farm were kept approximately under the same managemental conditions.

### Samples collection and examination

Fecal samples were collected from the rectum of each animal and placed in labeled Ziplock^®^ plastic bags. The samples were shipped in iceboxes to the Laboratory for microscopic examination. These samples were processed by both qualitative (floatation and sedimentation) and quantitative (modified McMaster egg counting) techniques as previously described (38–40).

### Feeding management of experimental animals

All experimental animals were kept under the same environmental conditions with a slight difference in the types of concentrate and fodder. Briefly, cattle and cattle-heifers were fed with a concentrate of Dairy Pellet (crude protein: 18–21%) of Maxim International Pvt. Ltd. and silage, while buffaloes and buffalo-heifers were fed with manually prepared concentrate (No recorded crude protein) containing a mixture of different food ingredients (cotton seed cake, rice bran, wheat bran, bread pieces, and mustard oil) and seasonal fodders.

### Anthelmintic and its efficacy

The average EPG of all animal groups before medication (day 0) is shown in **Tables 2**, **3**. Following these findings, animals were classified into control and treatment groups. The animals of treatment groups were medicated with albendazole (Valbazen 113.6 mg/ml; Pfizer Pharma, New York) at the rate of 10 mg/kg body weight. The efficacy was calculated upon average variation in egg reduction compared with day 0. Fecal egg count reduction testing (FECRT) was used to identify the helminths' resistance against albendazole. This test was applied till 60 days post-medication with 2 weeks intervals compared with the EPG count of infected animals before/after treatment (41). The weight gain of treated groups was subtracted from control groups to estimate the actual weight gain upon the anthelmintic appliance. The anthelmintic efficacy was estimated using the following formula:


$$\begin{array}{l} \text{ Effectiveness }=\;100\;\times \;(\text{Pre}-\text{treated}\\ EPG-Post-treated\;EPG)/\text{Pre}-\text{treated EPG}. \end{array}$$

### Economic evaluation of the farms

For the cost-benefit ratio, the considered input variables were the cost for treatment, feed, and labor while output variables were milk production and estimated live weight gain (42). Local market prices of these variables in each studied area were considered according to their region for this study. Briefly, the treatment cost of anthelmintic for adult cattle and buffalo was estimated to be US$ 1.2/animal, while for cattle-heifer and buffalo-heifer it was US$ 0.4 and 0.2/animal, respectively. The price and details of feed costs were estimated as previously described by Rashid et al. (2). Cost of input parameters including feed [concentrate (US$ 0.4/kg for cattle and 0.27 US$ 0.4/kg for buffalo) and fodder (silage of US$ 0.048/kg for cattle and seasonal fodder of 0.029 US$/kg for buffalo)], treatment and labor were encountered. On dairy farms, one labor was engaged for 10 adults or 20 heifers with a pay package of US$ 114.59/month (https://www.reference.com/geography/average-salary-pakistan-d487909030150b6f). Income consists of milk production (cattle for US$0.54/L and buffalo for 0.76/L) and lives weight (US$ 3.82/kg) gain was taken for the *cost*-*benefit ratio* (CBR). Live weight gain/loss of heifers was measured at days 0, 7, 15, 30, 45, and 60 according to the formula.


$$\begin{array}{l} \text{Income from variation in relative body weight}:\\ \left[\text{Live weight at last visit}-\text{Live weight at day }0\right]\;\times \\ Market\;price\;\left(kg\right) \end{array}$$

### Statistical analysis

Descriptive statistical and two-way ANOVA analysis was performed on EPG and body weight gain in control and treated groups using GraphPad Prism 7 for Windows (GraphPad Software, San Diego, California, USA, www.graphpad.com). The percentage of EPG reduction within 2 weeks interval was calculated with reference to pre-medication (days 0) while the increment in milk production was calculated from the control group and analyzed by Student *t*-test. Moreover, the value of CBR was calculated from the total income and cost. The threshold value of 5% was considered for all the statistical tests.

## Results

### Approved experimental protocol

Samples were collected according to instructions and guidelines approved by animal Ethics committee No. DR 1112.

### Infestation prevalence

A total number of eight types of helminth species were present in the studied animals. The most prevalent helminths were *Ostertagia* sp. (8.3, 5.0, 8.3, and 8.3%), *Strongyloides* sp. (8.3, 10.0, 8.3, and 8.3%) followed by *Oesophagostomum* sp., *Haemonchus* sp., *Trichostongylus* sp., *Moniezia* sp., *Toxocara vitulorum*, and *Fasciola* in cattle, cattle-heifer, buffaloes, and buffaloes-heifers, respectively. The overall prevalence of helminth infection in cattle and cattle-heifers was recorded to be 73.3 and 78.3%, respectively, whereas in buffaloes and buffalo-heifers it was 76.6 and 85.0%, respectively (p > 0.05). Specifically, a higher prevalence of *Haemonchus* sp. (10.0–13.3%) was observed in cattle and cattle-heifers, followed by *Fasciola* (10.0–11.6%) in buffaloes and buffalo-heifers, respectively while a lower prevalence of *Toxocara vitulorum* was observed in both animal species (cattle and buffaloes) (Table 1).

**Table 1:** Prevalence of helminth in cattle, cattle-heifers, buffaloes, and buffalo-heifers.

**Helminthes type**	**No. of cattle (%)**	**No. of cattle heifer (%)**	**No. of Buffalo (%)**	**No. of Buffalo heifer (%)**
*Fasciola*	3(5.0)	4(6.7)	6(10.0)	7(11.7)
*Ostertagia*	5(8.3)	3(5.0)	5(8.3)	5(8.3)
*Trichostrongylus*	3(5.0)	2(3.3)	4(6.7)	4(6.7)
*Oesophagostomum*	2(3.3)	3(5.0)	5(8.3)	3(5.0)
*Strongyloides*	5(8.3)	6(10.0)	5(8.3)	5(8.3)
*Toxocara vitolurum*	2(3.3)	1(1.7)	2(3.3)	3(5.0)
*Haemonchus*	6(10.0)	8(13.3)	–	–
*Moniezia*	4(6.7)	4(6.7)	4(6.7)	4(6.7)
*Mixed infection*	14(23.3	16(26.6)	15(25.0)	20(33.33)
**Total infected**	44	47	46	51
**Overall prevalence**	73.3	78.3	76.7	85.0

A total of 60 animals were taken for each group.

### Anthelmintic efficacy

The reduction of EPG on day 7 in treated cattle and cattle-heifers was 88.1 and 91.9% whereas in buffaloes and buffalo-heifers it was 92.0 and 93.8%, respectively. The highest EPG reduction rate observed on day 14 in treated cattle and cattle-heifers was 93.8 and 94.4% respectively. In buffaloes and buffalo heifers, the maximum reduction in EPG count was 95.2 and 97.8%, respectively on day 14. The reduction in EPG reached 73.9 and 70.9% in cattle and cattle-heifers, respectively while in buffaloes and buffalo-heifer reduction was 71.6 and 75.5% on 60 days of post-anthelmintic treatment. The anthelmintic was highly significant (*p* < 0.001) in helminth reduction with respect to the control group (Tables 2, 3).

**Table 2:** Descriptive analysis [mean EPG and standard error (SE)] with 95% confidence interval (CI) in control and medicated groups of cattle and cattle-heifers.

**Days**	**Cattle**						**Cattle-heifers**						***P-value***
	**Control (*****n*** = **20**			**Treatment (*****n*** = **20)**			**Control (*****n*** = **20**			**Treatment (*****n*** = **20)**			
	**Mean ±SE**	**Increment %**	**95% CI**	**Mean ±SE**	**Efficacy %**	**95% CI**	**Mean ±SE**	**Increment %**	**95% CI**	**Mean ±SE**	**Efficacy %**	**95% CI**	
0	690.0 ± 12.4		664.1–715.9	652.5 ± 40.3	-	568.1–736.9	742.5 ± 52.0		718.2–766.8	680.0 ± 41.4		593.3–766.7	< 0.0001
7	737.5 ± 13.0	6.9	710.3–764.7	77.5 ± 14.7	88.1	46.7–108.3	810.0 ± 70.0	9.1	777.3–842.7	55.0 ± 12.5	91.9	28.8–81.2	
14	785.0 ± 13.6	13.8	756.5–813.5	40.0 ± 10.6	93.9	17.7–62.3	867.5 ± 59.1	16.8	839.8–895.2	37.5 ± 8.0	94.5	20.7–54.3	
30	860.0 ± 12.4	24.6	834.1–885.9	55.0 ± 9.5	91.6	35.1–75.0	942.5 ± 63.4	26.9	912.8–972.2	70.0 ± 11.7	89.7	54.5–94.5	
45	902.0 ± 14.3	30.8	872.6–932.4	112.5 ± 8.8	82.8	94.1–130.9	1002.5 ± 61.7	35.0	973.6–1031.0	125.0 ± 10.6	81.6	102.9–147.1	
60	927.5 ± 10.6	34.4	905.4–949.6	170.0 ± 8.4	74.0	152.4–187.6	1042.5 ± 76.6	40.4	1007.0–1078.0	197.5 ± 12.8	71.0	170.7–224.3	
*P*-value (between group)	< 0.0001	

Anthelmintic efficacy was evaluated by fecal egg count reduction testing (FECRT) with reference to pre-medication in each group. Here n is the total number of animals, SE is the standard error, CI is confidence interval, and FECR is fecal egg count reduction rating. Fecal egg count of control and medicated animals cattle and cattle-calf were taken to find out the anthelmintic efficacy by fecal egg count reduction.

**Table 3:** Descriptive analysis [mean EPG and standard error (SE)] with 95% confidence interval (CI) in control and medicated groups of buffaloes and buffalo-heifers.

**Days**	**Buffaloes**						**Buffaloes –heifers**						***P-*value**
	**Control (*****n*** = **20)**			**Treatment (*****n*** = **20)**			**Control (*****n*** = **20**			**Treatment (*****n*** = **20)**			
	**Mean ±SE**	**Increment %**	**95% CI**	**Mean ±SE**	**Efficacy %**	**95% CI**	**Mean ±SE**	**Increment %**	**95% CI**	**Mean ±SE**	**Efficacy %**	**95% CI**	
0	737.5 ± 11.4	-	713.6–761.4	785.0 ± 50.8	-	678.7–891.3	755.0 ± 12.0	-	729.9–780.1	815.0 ± 50.3	-	709.8–920.2	< 0.0001
7	787.5 ± 14.0	6.8	758.2–816.8	62.5 ± 12.0	92.0	37.5–87.5	820.0 ± 12.3	8.6	794.4–845.6	50.0 ± 10.9	93.9	27.2–72.8	
14	805.0 ± 14.5	9.2	774.7–835.3	37.5 ± 8.0	95.2	20.7–54.3	847.5 ± 11.2	12.3	824.1–870.9	17.5 ± 6.6	97.9	3.8–31.2	
30	892.5 ± 12.7	21.0	865.9–919.1	130.0 ± 9.2	83.4	110.8–149.2	947.5 ± 10.6	25.5	927.7–967.3	45.0 ± 10.2	94.5	23.7–66.3	
45	942.5 ± 19.6	27.8	901.4–938.6	177.5 ± 9.2	77.4	158.2–196.8	1040.0 ± 11.2	37.8	1016.0–1064.0	147.5 ± 7.7	81.9	131.4–163.6	
60	1005.0 ± 15.8	36.3	972.0–1038.0	222.5 ± 9.9	71.7	201.7–243.3	1145.0 ± 12.0	51.7	1120.0–1170.0	200.0 ± 10.3	75.5	178.5–221.5	
*P*-value (between group)	< 0.0001

Anthelmintic efficacy was evaluated by fecal egg count reduction testing (FECRT) with reference to pre-medication in each group.

### Anthelmintic effect on production

**Figure 1:**
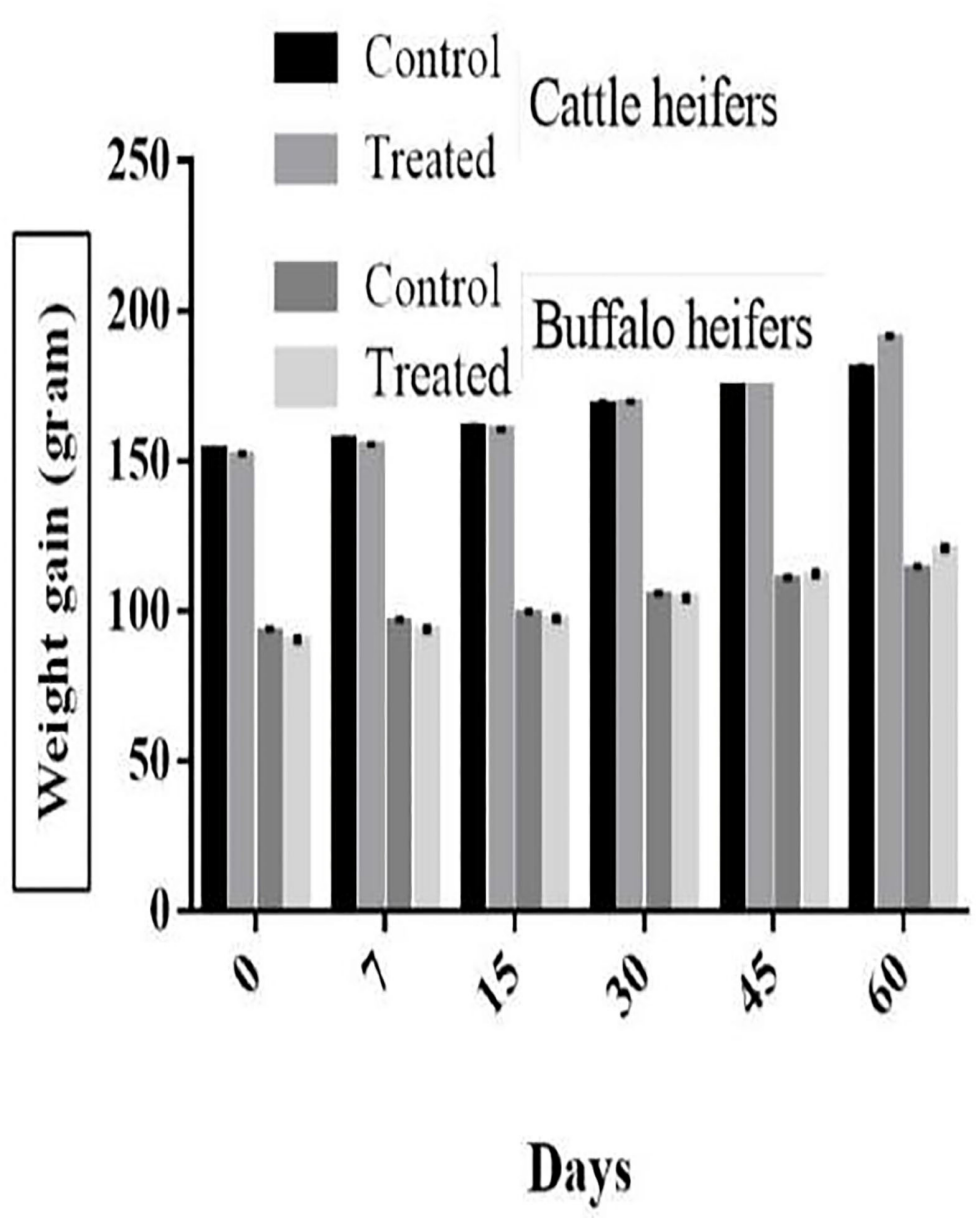
Anthelmintic effect on the weight gain of cattle and buffaloes heifers.

The average milk yield of control and treated cattle was 18.5 ± 0.36 and 19.3 ± 0.32 L/day, respectively (*p* > 0.05) with differences of 0.8L/day whereas in buffaloes it was 7.5 ± 0.22 and 8.2 ± 0.23 L/day (*p* > 0.05) with differences of 0.7 L/day (buffalo). The mean weight of cattle-heifers ranged from 151.4 to 153.0 kg/heifer while buffalo-heifers ranged from 89.9 to 92.8 kg/heifer at pre-medication (day 0). The weight gain in treated heifers after 60 days was significant (*p* < 0.05) compared with the control group. After 60 days of post-anthelmintic treatment, the mean weight gains in treated cattle-heifer and buffalo-heifers were recorded on days 0, 7, 15, 30, 45, and 60 with a total gain of 11.5 and 9.5 kg, respectively (Figure 1).

### Cost-benefit ratio

The CBR values indicate the ratio of income/loss per unit cost. The highest CBR value was recorded for the animal groups of treated adult cattle (2.515) followed by control adult cattle (2.423), treated adult buffalo (1.759), control adult buffalo (1.618), treated buffalo-heifer (1.351), treated cattle-heifer (1.056), control buffalo-heifers (0.935), and control cattle-heifers (0.748). Higher CBR values indicate farm profitability while lesser value shows a loss in the rearing of those animals (Table 4).

**Table 4:** Cost-benefit ratio (CBR) of treated and control groups.

**Species**	**Animal group**	**Animal type**	**Treatment cost**		**Per animal per day feed cost**					**Total cost and income for 60 days**				
			**Dose (ml)**	**Cost**	**Conc. Qty. (kg)**	**Cost**	**Fodder Qty. (kg)**	**Cost**	**Total feed cost**	**Labor cost**	**Total cost (Feed+Labor)**	**Qty. in kg/unit price (milk and weight)**	**income**	**CBR**
Cattle	Treated	Adult	50	1.2	6	2.4	28	1.34	224.4	23.0	248.6	1158.0/0.54	625.3	2.525
		Heifer	15	0.4	3	1.2	20	0.95	129.0	11.5	140.9	39.0/3.82	148.8	1.056
	Control	Adult	-	-	6	2.4	28	1.34	224.4	23.0	247.4	1110.0/0.54	599.4	2.423
		Heifer	-	-	3	1.2	20	0.95	129.0	11.5	140.5	27.5/3.82	105.1	0.748
Buffalo	Treated	Adult	50	1.2	8	2.14	35	1.0	188.4	23.0	212.6	492.0/0.76	373.9	1.758
		Heifer	10	0.2	3	0.8	15	0.435	74.1	11.5	85.8	30.4/3.82	115.9	1.351
	Control	Adult	-	-	8	2.14	35	1.0	188.4	23.0	211.4	450.0/0.76	342.0	1.618
		Heifer	-	-	3	0.8	15	0.435	74.1	11.5	85.6	21.0/3.82	80.0	0.95

Income and cost are mentioned in United State Dollars (USD). Here Conc. Qty is the concentrate quantity, ml is milliliter and CBR is the cost-benefit ratio.

## Discussion

The current study provides the data on the most prevalent helminth species (*Haemonchus* sp. in cattle and *Fasciola* sp. in buffaloes), mixed parasitic infection, AE, and CBR. In the field condition, a higher prevalence of fasciolosis (20%) in cattle and buffaloes in Toba Tek Singh, Pakistan was observed (43). A higher prevalence of 23.3, 26.67, 25 and 33.33% of mix infection was observed in cattle, cattle-heifer, buffaloes, and buffalo-heifers in the studied animals. Similarly, the prevalence of mixed infection was found at 7, 18, 9, and 23% in cattle, cattle-heifer, buffaloes, and buffalo-heifers, respectively in a study conducted in the same province similar to the current study. This previous study's finding (0.00–3.27) for the prevalence of *Oesophagostomum* was slightly different (3.3–8.3) from the current study (15). The similarity (mixed infections) and difference (*Oesophagostomum*) were due to the same study area and different feeding systems. The distribution of these parasites at dairy farms are mainly attributed to the contaminated fodder (44) and intermediate host. Moreover, since feed and water are available *ad libitum* to all free-moving animals at dairy farms (45), the incidence is likely to occur frequently. Generally, the mean EPG in heifers was higher than in adult animals pertaining to the higher susceptibility of heifers than immune-compromised adult animals (46). Moreover, the mean EPG in the buffaloes breed was higher than cattle breed representing their susceptibility or no previous anthelmintic treatment. The EPG in control groups increased with the passage of time due to the persistent and continuous expansion of intestinal parasites as already described by Saqib et al. (37). The maximum reduction in EPG was on the 14th day post-medication. However, a higher reduction in percentage was observed in buffaloes and buffalo-heifers indicating a reduced anthelmintic resistance as compared to cattle breeds (47) that are treated regularly favoring the establishment of anthelmintic resistance. The efficacy of anthelmintic was not 100% in any animal species/breed which might be the problem of drug resistance. It might be due to the regular or under-dose usage of the same group of medicine. Therefore, it is recommended to apply alternative treatment, and a combination of two groups (37). Generally, investors consider exotic cattle breeds over the buffaloes at dairy farms mainly due to a higher milk yield (2). In this study, the average increase in milk production in cattle and buffalo was 0.8 and 0.7 L/ day, respectively, which confirms previous studies undertaken in the Netherlands (48). This increase in milk production in cattle was lower than in buffaloes and this can be associated with less reduction of EPG in cattle in the local production system. The average weight of cattle-heifers was higher than buffalo-heifers at the same age which might be due to the improved genetic potential in the cattle breed. Owing to the increasing genetic profile, the first conception rate of cattle heifers is earlier at 14–16 months of age than buffalo heifers which are at 22 months of age (49, 50). The weight gain of treated buffalo and cattle-heifers was 500 and 650 g/day while in the control group it was 350 and 460 g/day, respectively, which is in accordance with the results of a previous study on a dairy farm in Pakistan and the Manawatu region of New Zealand (49, 51). Additionally, the CBR of treated animals was higher than that of the non-treated ones (37) in adult cattle followed by adult buffaloes, buffalo-heifers, and cattle-heifers. It is noteworthy, that infrastructure cost to control environmental conditions and auto-machine, which is necessary for exotic animal dairies was not considered. On the other hand, native buffaloes do not require extra management as they are fully acclimatized and adapted to the native environment. Considering this difference in managemental cost, the CBR of cattle will be considerably lower than buffaloes. It was also observed in this study that non-treated cattle-heifers and buffalo-heifers were not profitable, mainly due to their susceptibility to parasitic infections and extra requirements of feed at this stage of development, which can be interfered with by the existing parasites.

## Conclusions

Taken together, these studies indicate that anthelmintic treatment may positively impact EPG reduction and can cause an increase in milk production and gain in body weight in both cattle and buffaloes at studied dairies. Collectively, this improved efficiency of production favors the direct and positive cost-benefit ratio for dairy farmers.

## Data availability statement

The original contributions presented in the study are included in the article/supplementary material, further inquiries can be directed to the corresponding author/s.

## Ethics statement

The animal study was reviewed and approved by DR 1112. Written informed consent was obtained from the owners for the participation of their animals in this study.

## Author contributions

MR and AC performed experiments. MR, AC, and NZ drafted MS. TR and MTA did proofreading and correction. MIR did data analysis. AB provided sources for sampling and data history of animals. AA, AM, and MMA provide the funding and proofreading of MS. ME, MM, and GH did data curation. All authors contributed to the article and approved the submitted version.

## Funding

The work was financially supported by the researchers supporting project number (RSP2022R494), King Saud University, Riyadh, Saudi Arabia.

## Conflict of interest

The authors declare that the research was conducted in the absence of any commercial or financial relationships that could be construed as a potential conflict of interest.

The reviewer KL declared a shared affiliation with the author MTA to the handling editor at the time of review.

## Publisher's note

All claims expressed in this article are solely those of the authors and do not necessarily represent those of their affiliated organizations, or those of the publisher, the editors and the reviewers. Any product that may be evaluated in this article, or claim that may be made by its manufacturer, is not guaranteed or endorsed by the publisher.
